# Cigarette Smoke and Inflammation: Role in Cerebral Aneurysm Formation and Rupture

**DOI:** 10.1155/2012/271582

**Published:** 2012-12-13

**Authors:** Nohra Chalouhi, Muhammad S. Ali, Robert M. Starke, Pascal M. Jabbour, Stavropoula I. Tjoumakaris, L. Fernando Gonzalez, Robert H. Rosenwasser, Walter J. Koch, Aaron S. Dumont

**Affiliations:** ^1^Joseph and Marie Field Cerebrovascular Research Laboratory, Division of Neurovascular & Endovascular Surgery, Department of Neurological Surgery, Thomas Jefferson University, Philadelphia, PA 19107, USA; ^2^Department of Neurological Surgery, University of Virginia School of Medicine, Charlottesville, VA 22903, USA; ^3^Center for Translational Medicine and George Zallie and Family Laboratory for Cardiovascular Gene Therapy, Department of Medicine, Thomas Jefferson University, Philadelphia, PA 19107, USA; ^4^Division of Neurovascular & Endovascular Surgery, Department of Neurological Surgery, Thomas Jefferson University, 901 Walnut Street, 3rd Floor, Philadelphia, PA 19107, USA

## Abstract

Smoking is an established risk factor for subarachnoid hemorrhage yet the underlying mechanisms are largely unknown. Recent data has implicated a role of inflammation in the development of cerebral aneurysms. Inflammation accompanying cigarette smoke exposure may thus be a critical pathway underlying the development, progression, and rupture of cerebral aneurysms. Various constituents of the inflammatory response appear to be involved including adhesion molecules, cytokines, reactive oxygen species, leukocytes, matrix metalloproteinases, and vascular smooth muscle cells. Characterization of the molecular basis of the inflammatory response accompanying cigarette smoke exposure will provide a rational approach for future targeted therapy. In this paper, we review the current body of knowledge implicating cigarette smoke-induced inflammation in cerebral aneurysm formation/rupture and attempt to highlight important avenues for future investigation.

## 1. Introduction

Cigarette smoking is a major health hazard, with 5.4 million premature deaths worldwide every year and an average loss of 13 to 15 years of life expectancy [1]. Among the numerous serious health risks attributed to smoking, cerebral aneurysms stand as a major and potentially devastating clinical problem. Despite considerable advances in diagnostic methods, surgical techniques, and perioperative management, the outcome for patients with aneurysmal subarachnoid hemorrhage (SAH) remains poor, with mortality rates as high as 65% and morbidity rates in the range of 50% among survivors [2, 3]. Cigarette smoke (CS) is the most significant modifiable risk factor for cerebral aneurysm formation. Additionally, CS is a major risk factor for rupture with a hazard ratio reportedly as high as 3-4 [4, 5]. Up to 80% of patients who sustain an aneurysmal SAH have a history of smoking, and 50–60% are current smokers. Despite the strength of this association, the underlying pathogenic pathways remain largely unknown. A burgeoning but currently incomplete body of evidence suggests that vascular inflammation, a key component of cerebral aneurysm pathogenesis, may provide the common link between cigarette smoking and aneurysm formation and rupture. Accordingly, exposure to chemicals in cigarette smoke has consistently been shown to have a significant effect on various pathways of the immune/inflammatory response in the cerebrovascular system [6, 7]. This complex interplay between CS and vascular inflammation in cerebral aneurysm pathogenesis may represent an important target for future therapy. The present discussion critically evaluates the existing body of literature implicating active and passive cigarette smoking in aneurysm formation/rupture and attempts to highlight important avenues for future investigation.

## 2. Cigarette Smoke

CS is a complex and reactive mixture of some 5000 chemicals generated upon burning of the ingredients of tobacco. Some smoke components such as carbon monoxide, carbon dioxide, and nitrogen dioxide are gases. Others such as nicotine, phenol, polyaromatic hydrocarbons, and certain tobacco-specific nitrosamines are contained in the particulate phase which may also enter the bloodstream. The particulate phase of CS contains >10^17^ free radicals per g, and the gas phase contains >10^15^ free radicals per puff [8]. The radicals contained in the tar phase are long-lived (hours to months), whereas those associated with the gas phase have a shorter life span (seconds) [8]. Nicotine, carbon monoxide, reactive oxygen species (ROS), and acrolein are CS toxins with significant inflammatory and immunomodulatory potential [6].

Passive smoking from exposure to environmental tobacco smoke has been shown to increase the risk of cardiovascular disease including ischemic stroke [9, 10]. Moreover, it is estimated that passive smoking is the third leading preventable cause of death in the United States, behind active smoking and alcohol [9]. Despite the proven deleterious effects of passive smoking, data implicating environmental tobacco smoke exposure in aneurysm formation and rupture are lacking. An epidemiologic study by Anderson et al. [11] that included 432 cases of SAH matched to 473 SAH-free controls did not find an association between passive smoking and SAH. However, the study was underpowered to detect small risks of SAH in subjects exposed to passive smoking. Also, measurement of exposure to environmental smoke was limited only to the home and did not include the workplace or other areas, which may have underestimated the potential association between passive smoking and SAH.

## 3. Pathogenesis of Cerebral Aneurysms: An Overview

Accrued data suggest that aneurysm formation begins with endothelial dysfunction in response to alterations in flow and shear stress (e.g., arterial bifurcations) [12–14]. The endothelial dysfunction leads to compensatory responses that alter the normal homeostatic properties of the endothelium. Subsequent functional and morphological changes in the endothelium trigger a mounting inflammatory response in the vessel wall involving leukocytes, cytokines, adhesion molecules, immunoglobulins, complement, and many other key inflammatory components [15, 16]. An important aspect of this inflammatory reaction is the phenotypic modulation of vascular smooth muscle cells (VSMC) from a contractile and differentiated phenotype into a proinflammatory and dedifferentiated phenotype. Collectively, these changes lead to extracellular matrix remodeling by matrix metalloproteinases (MMP) with loss of the internal elastic lamina, thinning of the media, and aneurysm formation [17–19]. Continued inflammation, loss of VSMC with decreased collagen synthesis, and excessive extracellular matrix breakdown culminate in aneurysm rupture and SAH [20].

Recent reports suggest that one of the major contributing factors to cerebral aneurysm formation is the “genetic make-up” of the individual. The role of genetics is highlighted by the increased risk of cerebral aneurysms in first-degree relatives of SAH patients [4]. The prevalence of cerebral aneurysms is reportedly 2.3% in the general population, 4% in people with 1 affected first-degree relative, and 8% in those with 2 affected first-degree relatives [21]. The increased incidence with genetic diseases such as adult polycystic kidney disease, Ehler-Danlos type IV, and fibromuscular dysplasia also supports a genetic contribution to aneurysm formation and rupture [4]. Although several associations have been made between cerebral aneurysms and specific genetic polymorphisms, none of these associations have been consistently reproducible [22]. Candidate genes are those associated with vascular wall formation, response of the cerebral artery to increased stress, or those implicated in connective tissue disorders. A recent study that included 298 cases and 488 controls examined whether type III collagen variants contribute to the risk of sporadic intracranial aneurysms in the Chinese population [23]. The study found that the functional variant of COL3A1 conferred a 1.71-fold increased risk for cerebral aneurysms. The elastin locus has also been found to be implicated in aneurysm genesis, though definitive evidence is still lacking [24, 25]. Yasuno et al. [26] provided evidence for a significant association between endothelin receptor type A gene and cerebral aneurysms, while Khurana et al. [27] identified a set of endothelial NO synthase (eNOS) gene polymorphisms that were more prevalent among patients with ruptured versus unruptured cerebral aneurysms. Several other candidate genes have been investigated such as transforming growth factor-*β* receptors, elastase, polycystin, and fibrillin, with varying results [22, 28–30]. Most of the gene variants implicated in intracranial aneurysms are CS targets, and this will be reviewed in detail in this paper. Although the exact gene variants that are associated with intracranial aneurysms have yet to be found, smoking appears to greatly enhance their effect. This has been recently demonstrated by Deka et al. [31] who found that the variants on chromosomes 8q and 9p are associated with intracranial aneurysms and that the risk of aneurysm formation in patients with these variants is greatly increased with cigarette smoking. 

The risk of SAH is increased by the presence of symptomatic, large (>7 mm), and posterior circulation aneurysms [32]. Female sex, hypertension, excessive alcohol consumption, and cocaine abuse are important risk factors for SAH [33]. Vlak et al. [34] identified 8 trigger factors for aneurysmal rupture, namely, coffee consumption, cola consumption, anger, startling, straining for defecation, sexual intercourse, nose blowing, and vigorous physical exercise. A recent case-control study from 4 Australasian cities found that frequent intake of fat increases the risk of SAH while frequent use of skim or reduced-fat milk and fruit is protective against SAH [35]. Dietary antioxidants and soy products have also been found to be protective against the development of SAH in case-control studies, but prospective data are needed to confirm these findings [36, 37].

## 4. The Role of Atherosclerosis

The presence of atherosclerotic lesions in vessel walls is a significant feature of cerebral aneurysms [38, 39]. Small aneurysms typically demonstrate diffuse intimal thickening composed predominantly of proliferating VSMC whereas larger aneurysms have advanced atherosclerotic plaques with alternating layers of lipid-laden macrophages and mature VSMC. The observation that atherosclerotic lesions are found even in the smallest aneurysms and that progression of these lesions correlates positively with aneurysmal growth has led many investigators to hypothesize that atherosclerosis underlies cerebral aneurysm formation, growth, and rupture [38–41]. This is further corroborated by the fact that atherosclerosis is a crucial element in the causal pathway for aneurysms arising elsewhere in the body, particularly abdominal aortic aneurysms [42–44]. 

In a potential link to cerebral aneurysm formation, CS mediates various critical signaling pathways that underlie vascular inflammation in atherogenesis. In fact, CS results in endothelial dysfunction with impaired NO-dependant vasodilatory function and elevates various proinflammatory cytokines involved in atherosclerosis with subsequent recruitment and transendothelial migration of leukocytes [45–49]. Additionally, CS causes VSMC stimulation/proliferation and increases oxidative modification of low density lipoproteins (LDL) which are subsequently taken up by modified macrophages to form foam cells [45–49]. Most of these abnormalities are accounted for by free radical-mediated oxidative stress from CS exposure [48]. Taken together, these data indicate that the pivotal role of CS-induced inflammation in the initiation and progression of atherosclerosis may be a potential mechanism for cerebral aneurysm formation and rupture.

## 5. Cigarette Smoke and Hemodynamic Stress

Hemodynamic forces, particularly wall sheer stress, play a key role in cerebral aneurysm development [50–52]. Accordingly, cerebral aneurysms are most commonly found at locations that are exposed to major hemodynamic forces, namely, at arterial bifurcations, junctions, or acute vascular angles. Hemodynamic insult has been shown to cause rapid degradation of the internal elastic lamina followed by thinning of the media and outward bulging of the vessel wall [51, 53, 54]. These changes appear to be mediated by several key components of the inflammatory response which are upregulated in response to hemodynamic stress, including nuclear factor-*κ*B (NF-*κ*B), interleukin 1*β* (IL1*β*), nitric oxide (NO), and MMP [12, 15, 54]. 

Wall shear stress is directly proportional to flow velocity and blood viscosity and inversely to arterial diameter. Thus, CS can significantly increase wall shear stress, as it is known to increase blood viscosity and blood volume [7, 55]. Singh et al. [56] sought to elucidate the mechanisms by which cigarette smoking leads to cerebral aneurysm formation. Using a three-dimensional model, the authors elegantly demonstrated that smoking was associated with an incremental increase in wall shear stress at the site of aneurysm initiation (secondary to an increase in blood volume) and concluded that this finding could provide the missing link between CS exposure and aneurysm formation. Aside from increased blood viscosity and blood volume, CS may also raise wall shear stress by inducing cerebral vasoconstriction. The vasoconstrictive effects of CS and nicotine have been well documented and are usually attributed to the inhibition of eNOS and impaired NO signaling, a critical pathway for regulating cerebrovascular tone [57–59]. In a well-conducted study, Gerzanich et al. [60] found that chronic nicotine exposure blunts cerebral vasodilation by NO and demonstrated that the underlying mechanism involved a blockade of normal NO-mediated downregulation of calcium channels in cerebral VSMC. 

The endothelin system, a crucial pathway associated with hemodynamic stress, is another possible mechanism through which CS may lead to aneurysm formation. The expression of endothelin type B receptors in response to mechanical stretch plays a critical role in cerebral aneurysm pathogenesis, and its inhibition was shown to suppress aneurysm progression [61–63]. Indeed, CS directly upregulates endothelin type B receptors in cerebral arteries through activation of key intracellular inflammatory signal molecules, namely, mitogen-activated protein kinases (MAPK) and the NF-*κ*B signal pathway [64, 65]. 

Collectively, these data suggest a close relationship between CS exposure, hemodynamic stress, and the downstream inflammatory reaction leading to cerebral aneurysm development. 

## 6. Cigarette Smoke and Endothelial Dysfunction

Endothelial dysfunction is a hallmark of cerebral aneurysm biology [15, 66–68]. The endothelium responds to hemodynamic stress with a series of physiological changes that increase its procoagulant and vasoconstrictive properties, leukocyte adhesion/migration, and the production of cytokines and growth factors. Using vascular corrosion casts, Jamous et al. [54] demonstrated that changes in the morphological characteristics of endothelial cells were the earliest changes in the process of aneurysm formation. Later, the same group added that endothelial cell injury, as evidenced by the loss of eNOS expression at the apical intimal pad, was the earliest pathological change in the process, triggering an inflammatory reaction characterized by macrophage infiltration, proliferation of VSMC, and proteolytic degradation of the vessel wall [12]. Along these lines, Tada et al. [67] noted early endothelial damage with interendothelial gaps at the site of cerebral aneurysm and demonstrated that the disruption of endothelial tight junctions was associated with the migration of macrophages into aneurysm walls. Aoki et al. [69, 70] brought further evidence of the critical role of endothelial dysfunction in CA formation, reporting several key changes in endothelial cells in response to high shear stress, including increased activity of NF-*κ*B, monocyte chemoattractant protein-1(MCP-1), and prostaglandin E2 pathway.

 The ability of CS to induce endothelial dysfunction in cerebral arteries has been well documented [48, 65, 71–74]. Lipid-soluble smoke particles are toxic to cultured endothelial cells and reduce endothelium-dependent relaxation in cerebral arteries [59, 72, 73, 75]. Likewise, CS was shown to induce endothelial apoptosis by activating caspase-3 [76]. CS-induced endothelial dysfunction seems to be primarily caused by accelerated inactivation of NO (an important vasodilator substance critical to the normal homeostasis of the endothelium) due to increased production of ROS [71]. The mechanism appears to involve several key enzyme systems, including NADH/NADPH oxidase and xanthine oxidase, both of which significantly contribute to ROS formation [71, 77–79]. As such, Fang et al. [78] showed that impairment of NO-dependent vasodilatation in cerebral arteries of nicotine-treated rats was caused by the increased formation of superoxide anions and noted reversal of these changes after inhibition of NADPH oxidase by apocynin. Elevated C-reactive protein (CRP) levels caused by CS can also promote endothelial dysfunction by lowering the production of NO and reducing its bioactivity [80]. Aside from the NO system, CS-induced endothelial dysfunction may also involve the angiotensin (AT) system, as AT1-receptor blockade was found to prevent CS-related impairment of endothelium-dependent vasodilation [81]. 

Endothelial NO synthase (eNOS) protects arterial walls from vascular inflammation by relieving hemodynamic stress via NO production [82]. Based upon this premise, Aoki et al. [83] examined the role eNOS in cerebral aneurysm pathogenesis and reported a protective role of eNOS against aneurysm formation and cerebrovascular inflammation. Importantly, nicotine in CS was shown to suppress this protective role of eNOS through the production of superoxide anions [84, 85]. Moreover, CS increases the expression of inducible NOS (iNOS) [86], which is activated in response to inflammatory stimuli and contributes to various inflammatory diseases [82]. This is particularly important with regard to cerebral aneurysm because induction of iNOS is associated with an increase in aneurysm size and apoptotic cell death [87]. Similarly, suppression of iNOS with aminoguanidine, a relatively selective inhibitor of iNOS, was shown to decrease the incidence of aneurysms in rats [88]. Thus, it appears that CS can affect cerebral aneurysm formation and progression by modulating the expression of key endothelial enzymes involved in cerebrovascular inflammation.

The proinflammatory activity of CS at the endothelial level further extends to the expression of several vascular adhesion molecules by endothelial cells that promote the adherence of circulating inflammatory cells to the luminal endothelial surface followed by their migration and the resultant vascular inflammatory response [89, 90]. Moreover, nicotine was shown to affect endothelial tight junctions by decreasing the expression of ZO-1, occludin, cadherin, and adherent junctional proteins [91, 92] which, as discussed above, leads to the migration of macrophages and other inflammatory cells into cerebral aneurysm walls. Lastly, increased levels of matrix-degrading and proinflammatory changes including chemokines, cytokines, and STAT3 (inflammatory regulator) have been found in endothelial cells exposed to CS [89, 90, 93–95]. Taken together, experimental studies demonstrate that CS induces a proinflammatory activation of brain endothelial cells, disrupting their normal function and initiating CA formation.

## 7. Effect of Cigarette Smoke on VSMC

A solid body of evidence suggests that VSMC constitute an integral part of the pathways leading to cerebral aneurysm formation and rupture. As in atherosclerosis, VSMC migrate into the intima of cerebral aneurysm walls where they proliferate and produce myointimal hyperplasia [38, 96]. More importantly, VSMC undergo a phenotypic modulation from a differentiated phenotype concerned primarily with contraction (characterized by expression of contractile genes/proteins such as smooth muscle-myosin heavy chain (SM-MHC) and smooth muscle-*α*-actin) to a proinflammatory/promatrix remodeling phenotype characterized by the increased expression of cytokines, MMP, and ROS [97–99]. This phenotypic modulation is mediated, at least in part, by the transcription factor KLF4 [100], and seems to occur under the influence of various inflammatory stimuli including interleukin-1b (IL-1b) [101], platelet-derived growth factor (PDGF) [102], oxidized phospholipids [103], tumor necrosis factor-*α* (TNF-*α*) [104], Ets-1 [105], and AT II [106]. A later event involves the loss of VSMC from cerebral aneurysm walls with resultant thinning of the media and aneurysm rupture.

 CS was shown to promote several of the above-described changes involving VSMC. Several studies have reported that CS exhibits a direct effect on VSMC chemotaxis and proliferation [107–112]. As such, Chen et al. [113] demonstrated that CS extract promotes human VSMC proliferation as well as the expression of adhesion molecules and IL-6 through a NF-*κ*B-dependent pathway. We have investigated the role of CS in producing phenotypic modulation and inflammation in cerebral VSMC in rats [86]. We found that CS directly produced marked phenotypic modulation with decreased expression of VSMC marker genes and myocardin while markedly increasing expression of inflammatory/matrix remodeling genes (MMP, MCP-1, IL-1, and TNF-*α* expression). Selective inhibition of KLF4 reversed this profound phenotypic modulation, highlighting the pivotal role of KLF4 in this process. These observations provided crucial evidence of a direct role of CS in the phenotypic modulation of VSMC in the walls of cerebral aneurysms. Lastly, CS extract was shown to be toxic to cerebral VSMC through calcium-induced cell injury and cell death from upregulation of VSMC calcium channels as elegantly demonstrated by Gerzanich et al. [60] in cerebral arterioles of rats. Collectively, these data indicate that the early and late changes exhibited by VSMC during cerebral aneurysm formation, progression, and rupture are at least partly triggered by CS exposure.

## 8. Cigarette Smoke and Inflammatory Mediators

Several cytokines are increased in response to CS exposure [89, 114–119]. Specifically, smokers have higher levels of IL-1*β*, TNF*α*, and IL-6 [89, 114–120], all of which significantly contribute to the pathobiology of cerebral aneurysm. In fact, IL-1*β* is thought to be a key inflammatory mediator in cerebral aneurysm as disruption of the IL-1 gene decreased the incidence of mature aneurysms in a mouse model [121]. IL1*β* acts primarily through activation of NF-*κ*B in VSMC of cerebral aneurysm, reducing the biosynthesis of collagen and promoting apoptotic cell death [121, 122]. Likewise, TNF*α* was shown to induce apoptosis of VSMC in cerebral aneurysm walls while also activating MMP and vessel wall degradation [123–125]. IL-6 may also have an important role in cerebral aneurysm pathogenesis according to several genetic case-control studies. A recent meta-analysis of 8 genes and 13 polymorphisms in approximately 20,000 individuals found a strong association between IL-6 gene polymorphism and cerebral aneurysm [126].

 Another mechanism through which CS may induce/amplify the inflammatory reaction in cerebral aneurysm walls is through the modulation of NF-*κ*B expression. As such, increased activation of NF-*κ*B and its downstream inflammatory pathways were found in endothelial cells [89], VSMC [65, 113], and mononuclear cells [127] of smokers. NF-*κ*B activation is followed by increased expression of adhesion molecules, migration of leukocytes (macrophages in particular), and increased degradation of the ECM [65, 69, 89, 113, 127]. In a study by Aoki et al. [69], NF-*κ*B was activated in cerebral arterial walls in the early stage of aneurysm formation with upregulated expression of downstream genes, and its selective inhibition prevented macrophage infiltration and aneurysm formation. Thus, NF-*κ*B, a seemingly important target of CS, plays a crucial role in the initiation of cerebral aneurysm development by inducing inflammatory genes related to macrophage recruitment and activation. Another critical regulator of macrophage activation in cerebral aneurysms, also induced by CS, is MCP-1 [86, 128–131]. The importance of this factor was highlighted in animal studies where knockout of MCP-1 led to decreased CA formation, decreased macrophage infiltration, and decreased expression of MMP [128, 129].

There is a close relationship between CS exposure, macrophage activation, and MMP release. Indeed, Hossain et al. [89] found that CS induces macrophage differentiation in cerebral arteries with a marked enhancement in the release of IL-1*β*, TNF-*α*, and MMP-2/9. Likewise, increased levels of MMP and macrophages along with decreased levels of tissue inhibitor of metalloproteinases (TIMP) and elastin were found in carotid vessels of smokers as compared to nonsmokers [132]. Similarly, Vikman et al. [133] observed enhanced expression of MMP13 in CS-exposed cerebral arteries through activation of the MAPK inflammatory signaling pathway. We have also demonstrated that CS extract stimulates MMP in rat cerebral VSMC *in vitro* and in carotid smooth muscle cells *in vivo* [86]. This increased activity of MMP could well explain the higher incidence of SAH in smokers, as degradation of the ECM by MMP (which weakens the aneurysm wall) is indisputably a key event in cerebral aneurysm rupture [15, 20, 134]. Accordingly, serum MMP-9 levels increase in SAH patients and normalize by day 12 after-SAH, suggesting an intimate relationship between MMP-9 and aneurysm rupture [135]. Indeed, plasma MMP-9 is mainly dependent on neutrophil release (gelatinase granules) in human, and neutrophil serine protease release is one of the main determinants of aneurysmal rupture [136]. The inappropriate activation of MMP in cerebral arteries is not the only mechanism through which CS leads to cerebral aneurysm wall fragility. In fact, CS reduces the expression of prolyl-4-hydroxylase, a key enzyme in arterial wall collagen metabolism, resulting in reduced collagen synthesis and a thin vulnerable aneurysm wall [137]. Taken together, these data indicate that CS activates key inflammatory mediators that lead to macrophage activation, release of MMP, and decreased collagen synthesis, ultimately resulting in cerebral aneurysm thinning and rupture.

## 9. ROS and Cigarette Smoke

ROS are a major mediator of various inflammatory cascades. Free radical-mediated oxidative stress is thought to be a pivotal step for the development of cerebral aneurysm. In a well-conducted animal study by Aoki et al. [138], ROS-producing gene, p47phox, was upregulated in infiltrating macrophages with suppression of ROS-eliminating genes, suggesting that ROS overproduction occurred in aneurysm walls. Furthermore, cerebral aneurysm formation and inflammation in aneurysm walls were markedly suppressed by p47phox gene deletion or edaravone, a powerful free radical scavenger. As discussed above, CS is a major source of free radicals which could arise from (a) the gas or tar phase of CS, (b)circulating or in situ-activated leukocytes,and (c) endogenous sources of ROS, that is, uncoupled eNOS, xanthine oxidase, and the mitochondrial electron transport chain [48, 139]. Many of the abnormalities observed in cerebral aneurysm including endothelial dysfunction, proinflammatory reactions, adhesion molecule expression, VSMC proliferation, apoptosis, MMP activation, and tissue destruction may be at least partly explained by the effects of increased oxidative stress [8, 45, 140–142]. Accordingly, antioxidants have been shown to improve or reverse the proinflammatory attributes associated with CS [143–145]. Thus, ROS may constitute an integral part of the inflammatory reaction leading to CA formation and rupture in smokers. 

## 10. Cigarette Smoke and Thrombosis

CS predisposes to systemic and cerebral thrombosis [146–148]. Nicotine increases plasminogen activator inhibitor-1 via protein kinase C dependant pathway in human brain-derived endothelial cells [147]. Additionally, increased levels of tissue factor (TF), a key factor in thrombogenesis, and decreased levels of TF pathway inhibitor-1 are associated with CS [149, 150]. This predisposition to thrombosis is particularly relevant with regard to cerebral aneurysm because the presence of an organizing luminal thrombus was identified as a predictor of aneurysm rupture [15, 145]. Likewise, partial or total deendothelialization, a prerequisite for thrombus formation, is intimately associated with human cerebral aneurysm rupture [13, 16]. In fact, following endothelial injury and deendothelialization (from CS, hemodynamic stress, etc.), exposure of subendothelial prothrombotic structures initiates a coagulation cascade that leads to thrombus formation. Subsequently, erythrocytes and platelets become trapped in the fibrin meshwork with expression of chemotactic factors and macrophage/neutrophils infiltration [15]. Erythrocyte lysis, complement activation, and oxidative stress ultimately result in further breakdown of the cerebral aneurysm wall by MMP [15, 151]. Thus, by promoting the formation of a luminal thrombus, CS may further amplify the inflammatory and destructive reaction in aneurysmal walls predisposing to aneurysm rupture. 

## 11. Conclusion

**Figure 1:**
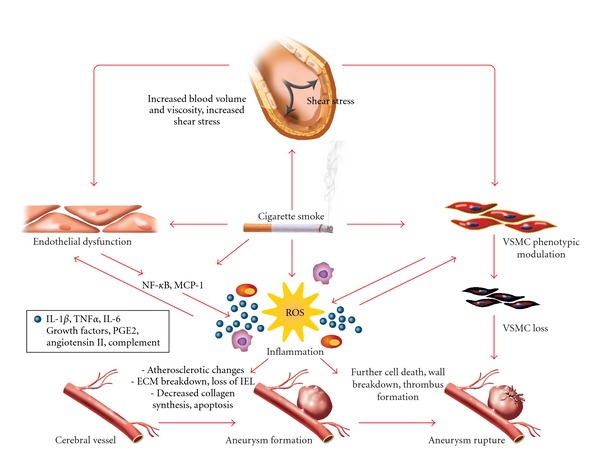
CS-associated inflammatory response in CA walls. CS increases wall shear stress in cerebral vessels and causes endothelial dysfunction with VSMC proinflammatory phenotypic modulation. The resultant inflammatory response implicates several inflammatory cells and mediators (ROS in particular) and leads to extracellular matrix remodeling and subsequent aneurysm formation. Further CS-induced matrix breakdown, cell death, and formation of an organizing thrombus eventually culminate in CA rupture. IEL: internal elastic lamina.

There is a complex yet interesting interplay between CS exposure, vascular inflammation, and cerebral aneurysm formation and rupture. CS appears to affect every step in the cascade of events leading to SAH from hemodynamic stress and endothelial dysfunction to aneurysm wall weakening and rupture (Figure 1, Table 1). The mechanisms described in this paper could explain why smokers are at increased risk of SAH. Further characterization of the role of CS-induced inflammation in cerebral aneurysm is needed to fill the considerable gaps in our current knowledge. Such knowledge could pave the way for the development of future targeted therapy. 

## Figures and Tables

**Table 1:** Summary of major pathways and inflammatory mediators affected by cigarette smoke exposure and involved in aneurysm formation.

Major pathways	Major inflammatory mediators
Atherosclerosis	Oxidized LDL
	NO
	MCP1
	IL1*β*
	ROS
	TNF*α*
	IL1*β*, IL-6
	Growth factors
	Selectins and adhesion molecules

Hemodynamic stress (cerebral vasoconstriction, increased blood viscosity, and volume)	eNOS
	Endothelin type B receptors
	NF-*κ*B

Endothelial dysfunction	NO
	ROS
	CRP
	Angiotensin
	eNOS
	iNOS
	Several chemokines and cytokines
	STAT3

VSMC proinflammatory, promatrix remodeling phenotypic modulation, apoptotic cell death	NF-*κ*B
	KLF4
	Calcium channels

Chronic inflammatory reaction, vessel wall remodeling and damage, apoptotic cell death	IL-1*β*, TNF*α*, and IL-6
	NF-*κ*B
	MMP and TIMP
	MCP-1
	MAPK
	ROS
	NO
